# Microorganisms’ colonization and their antibiotic resistance pattern in oro - tracheal tube

**Published:** 2013-06

**Authors:** Alireza Abdollahi, Saeed Shoar, Nasrin Shoar

**Affiliations:** 1 Division of Pathology, Imam Khomeini Hospital, Tehran University of Medical Sciences Tehran, Iran; 2 Department of Surgery, Shariati Hospital, Tehran University of Medical Sciences, Tehran, Iran; 3 School of Medicine, Kashan University of Medical Sciences, Kashan, Iran

**Keywords:** Nosocomial Infection, Microorganism Colonization, Antibiotic Resistance, Tracheal Intubation

## Abstract

**Background and Objectives:**

Recently, nosocomial infections have been discussed as a critical issue among intubated patients leading to significant morbidity and mortality. Hence, the pattern of microbiological colonization and antibiotic resistance are much valuable in this regard. We aimed to investigate the pattern of microorganism colonization and antibiotic resistance in patients with endotracheal tube or tracheostomy to propose a proper empirical antibiotic therapy in this setting.

**Materials and Methods:**

This cross sectional study was conducted among 880 patients admitted in Imam Khomeini hospital between 2008 and 2011 who were subsequently intubated or underwent tracheostomy due to insufficient self ventilation. Samples for microbiological cultures were obtained after extubation and then sent to the central laboratory for further assessment. Antibiograms and microbiological cultures were obtained for each sample.

**Results:**

Of 880 patients enrolled in this study, 531 (60.3%) were male and 349 (39.7%) were female. Nineteen different organisms were isolated including *Acinetobacter* (213, 24.2%), *Pseudomonas aeruginosa* (147, 16.7%), *Staphylococcus aureus* (106, 12%), *Proteus mirabilis* (90, 10.2%), and other organisms (324, 36.8%). Antibiotic resistance was mainly seen in *Acinetobacter* (ciprofloxacin, ceftazidim, cefepim, and penicillin), *S. aureus* (imipenem) and *Klebsiella* (pipracillintazobactam and ampicillin-sulbactam).

**Conclusion:**

This study represents the most common microorganisms colonizing tracheal tube of hospitalized patients and their pattern of antibiotic resistance. *Acinetobacter* was the most common microorganism isolated from endotracheal tube. Hence, it may be possible to initiate the empiric antibiotic treatment before the results of culture are become available. Ciprofloxacin was also the most prevalent antibiotic revealing resistant pattern. Moreover, most of the microorganisms were sensitive to imipenem and pipracillin-tazobactam.

## INTRODUCTION

Nosocomial infection is a frustrating and budget consuming issue and due to the increased time of hospitalization it imposes a heavy burden on health care resources (1). One of the most important types of this infection is pneumonia which commonly occurs in relation to the endotracheal intubation and mechanical ventilation named ventilation associated pneumonia (VAP) (2–5). Patients with mechanical ventilation have an increased risk for respiratory tract infection because the tube which has been inserted into the trachea reduces the clearance of bacteria and increases the leakage of secretion around the cuff of the tube and disable the cilliary tract by damaging to it (6). Because of decreased salivary secretion, colonization of oropharynx with Gram negative bacteria is also probable (7, 8).

In the literatures, the incidence of respiratory tract infection in relation to intubation and/ or mechanical ventilation has been reported to vary between 4% and 28% and this rate has been thought to be 21 times higher than in patients without endotracheal tube (9–13). Various studies have proposed different causative microorganism as the most common etiology for intubation related respiratory infections including *Pseudomonas aeruginosa, A. baumannii*, and methicillin resistant *Staphylococcuus aureus* (MRSA) or *S. aureus* in children (9, 14–19). The concerns related to the nosocomial infections are exacerbated by the presence of antibiotic resistant bacteria which increases morbidity rate and the associated costs (20). Extensive use of fluoroquinolones has lead to alterations in susceptibility patterns of microorganisms (10, 12, 13, 17). Furthermore, inappropriate prescription of broad-spectrum antimicrobial agents has risen in the last decades with macrolides, fluoroquinolones, and third-generation cephalosporins at the top of the list (14–16, 19, 20).

Our study hence aimed to determine the type of bacterial colonization and antibiotic sensitivity and resistance in patients with endotracheal intubation or tracheostomy in a tertiary care referral center to facilitate initiation of proper empirical antibiotic treatment in these patients.

## MATERIALS AND METHODS

Through a cross-sectional study in Imam Khomeini Hospital affiliated to Tehran University of Medical Sciences, 880 patients with positive culture of samples obtained from endotracheal tube or tracheostomy were enrolled in our investigation between 2008 and 2011. Patients had been intubated to maintain an airway, to be assisted by mechanical ventilation as a result of respiratory failure or just elective ventilation. Clinical impressions for necessitation of intubation or extubation were made by the responsible physicians based on clinical presentation of the patient and then endotracheal intubation (ET) was performed under a sterile technique; also extubation was done if the patients did assure clinically to be able to ventilate on his own without developing further complications. Patients with previous pneumonia or other airway infections were excluded from the study. Extubated ETs were cut by a sterile blade and the samples were sent to the microbiological laboratory for organism isolation and microbiological culture (for 48 to 72 hours) and obtaining an antibiogram. The isolated colony was then confirmed for type of microorganism by PCR (21). Antibiotic susceptibility was also evaluated by disc diffusion method on Mueller Hinton agar according to the guidelines of Clinical and Laboratory Standards Institute (CLSI) (22). Positive and negative control was used for antibiogram.

The samples were obtained from patients admitted to 3 wards of our hospital including neonatal ICU (NICU), ICU and emergency room (ER). Patients or their relatives on behalf of them filled an informed consent to enroll the study. Research Ethics Committee of TUMS approved the study protocol and the study was then conducted in a period of 3 years.

Data were analyzed by Statistical Package for Social Sciences (SPSS, version 16, Chicago, Inc) and the values are reported as number (%).

## RESULTS

A total of 880 patients were enrolled in our study including 531 male (60.3%) and 349 female (39.7%). Nineteen different microorganisms were isolated during the study including *Acinetobacter* (213, 24.2%), *Pseudomonas aeruginosa* (147, 16.7%), *Staphylococcus aureus* (106, 12%), *Proteus mirabilis* (90, 10.2%), and the remainder organisms which are summarized in Table 1. The most common organism in both genders was *Acinetobacter*. The most common organisms at different wards were as follows: *Acinetobacter* in ICU ward, *Entrococcus* in NICU, and *Staphylococcus aureus* in ER. In patients under the age of 12, *Klebsiella* was the most common organism while in those over the age of 12, *Acinetobacter* was the highest prevalent (Table 2). In terms of antiobiograms, 82% of *Acinetobacter*, 35.1% of *Staphylococcus aureus*, 33.3% of *Klebsiella* and 55.1% of *Proteus mirabilis* were resistant to ciprofloxacin (Table 3). These organisms were resistant to ceftazidime in 97.4%, 80%, 85.7% and 59.1% of the cases, respectively and resistant to imipenem in 7.4%, 18.2%, 1.8% and 8.1% of the cases, respectively. In addition, 100% of *Acinetobacter*, 77.8% of *Escherichia coli*, 75% of *Klebsiella* and 88.9% of *Proteus mirabilis* were resistant to cefepime. On the other hand, 91.7% of *Staphylococcus aureus*, 100% of *Acinetobacter* and 88.9% of *Staphylococcus epidermidis* were resistant to penicillin.

**Table 1:** Prevalence distribution of organisms isolated from different wards of the hospital.

Type of Organisms	Male	Female	<12 years (NICU)	>12 years
***Acinetobacter***	123*(23.3%)	90*(25.8%)	1 (3%)	212*(25%)
***Pseudomonas***	94 (17.7%)	53 (15.2%)	4 (12.1%)	143 (16.9%)
***S. aureus***	57 (10.7%)	49 (14%)	4 (12.1%)	102 (12%)
***Proteus mirabilis***	65 (12.2%)	25 (7.2%)	1** (3%)	89 (10.5%)
***Klebsiella***	39 (7.3%)	28 (8%)	6*(18.2%)	61 (7.2%)
***Entrobacter***	36 (6.8%)	20 (5.7%)	1** (3%)	55 (6.5%)
***E. coli***	32 (0.6%)	22 (.3%)	_	54 (6.4%)
***Citrobacter***	18 (3.4%)	10 (2.9%)	1** (3%)	27 (3.2%)
***Stenotrophomonas***	12 (2.3%)	10 (2.9%)	3 (9.1%)	19 (2.2%)
***Prodentia***	13 (2.4%)	6 (1.7%)	_	_
***Streptococcus viridans***	7 (1.3%)	9 (2.6%)	_	_
***S. epidermidis***	7 (1.3%)	5 (1.4%)	3 (9.1%)	9 (1.1%)
***Entrococcus***	4 (0.8%)	7 (0.2%)	5 (15.2%)	6 (0.7%)
***Seratia***	8 (1.5%)	2 (0.6%)	_	10 (1.2%)
***Candida albicans***	6 (1.1%)	2 (0.6%)	_	8 (0.9%)
***S. hemolyticus***	2 (0.4%)	5 (1.4%)	_	7 (0.8%)
***Proteus vulgaris***	6 (1.1%)	1** (0.3%)	_	7 (0.8%)
***Alcalginis***	1** (0.2%)	5 (1.4%)	4 (12.1%)	2 (0.2%)
***Salmonella***	1** (0.2%)	_	_	1** (0.1%)
**Total**	531 (60.3%)	349 (39.7%)	33 (4%)	847 (96%)

* Most common

** Least common

**Table 2:** Differentiation of organisms based on age and sex.

Type of Organism	ICU (%)	NICU (%)	ER (%)	Total
***Acinetobacter***	205*(38.7)	1 (3.4)	5 (38.5)	213*(24.2)
***Pseudomonas***	131 (17.1)	4 (13.8)	1**(7.7)	147 (16.7)
***S. aureus***	95 (10.3)	4 (35.3)	7*(53.8)	106 (12)
***Proteus mirabilis***	89 (12.5)	1 (3.4)	3*(75)	90 (10.2)
***Klebsiella***	62 (5.9)	6 (63.8)	1 **(25)	67 (7.6)
***Entrobacter***	54 (4.6)	1**(3.4)	_	56 (6.4)
***E. coli***	53 (6.7)	_	_	54 (6.1)
***Citrobacter***	24 (3.6)	1**(3.4)	_	28 (3.2)
***Stenotrophomonas***	19 (2.1)	3 (10.3)	_	22 (2.5)
***Prodentia***	19 (2.9)	_	_	19 (2.2)
***Streptococcus viridans***	3 (0.5)	_	_	16 (1.8)
***S. epidermidis***	9 (0.8)	3 (31.9)	_	12 (1.4)
***Entrococcus***	6 (0.6)	5*(17.2)	_	11 (1.2)
***Seratia***	10 (1)	_	_	10 (1.1)
***Candida albicans***	8 (0.6)	_	_	8 (0.9)
***S. hemolyticus***	7 (0.8)	_	_	7 (0.8)
***Proteus vulgaris***	7 (1.1)	_	_	7 (0.8)
***Alcalginis***	2**(0.2)	4 (13.8)	_	6 (0.7)
***Salmonella***	1** (0.2)	_	_	1**(0.1)
**Total**	**837**	**33**	**17**	**880 (100)**

* Most common

** Least common

**Table 3:** Prevalence of resistance to antibiotics.

Type of Antibiotic	Ciprofloxacin	Ceftazidime	Imipenem	Cefepime	Pipracillin-tazobactam	Ampi-sulbactam	Penicillin
***Acinetobacter***	82%	97.4%	7.4%	100%	6.3%	27%	100%
***S. Aureus***	35.1%	80%	18.2	-	-	-	91.7%
***Proteus mirabilis***	55.1%	59.1%	8.1%	88.9%	5.6%	55.6%	-
***Klebsiella***	33%	85.7%	1.8%	75%	14.3%	55%	-
***E. coli***	-	-	-	77.8%	-	-	-
***S. epidermidis***	-	-	-	-	-	-	88.9%

Antibiotic resistance and susceptibility pattern in general and categorized according to the type of isolates are presented in details in Table 4.

**Table 4:** Sensitivity to antibiotics in two organisms.

Microorganisms	Vancomycin	Cloxaciline
*S. aureus*	99%	33%
*Staph epidermidis*	100%	100%

## DISCUSSION

Infections are among the most important and the leading cause of mortality and morbidity in ICU. Endo-tracheal tubes are susceptible to infection and therefore it is important to be aware of the relevant factors and responsible organisms to take prompt action. The findings of this study would be helpful in selection of appropriate antibiotics. In this study, all the positive colonies obtained from the ET or tracheostomy cases were considered. The study by Simoni *et al*. (23) showed that 100% of samples from airway prosthesis are positive in culture; however, other studies have reported a positive culture rate between 0% and 33% in obtained samples from airway tubes. Cardinosa *et al*. have reported a positive culture result in 89% of their samples (24). The variation could be explained by the technique of intubation, clinical and individual characteristics of study population, colonization during intubation or lack of sufficient precautions for intubation due to the high work load in an emergency setting (25).

In our study, Gram negative bacteria were the most common isolated organisms including *Acinetobacter* and *Pseudomonas aeruginosa* which is in the same line with the study by Nardi *et al*. (36). However, in Cardinosa *et al*. study, during the first 24 hours of admission, Gram positive bacteria were the most common cause of infection and this could be as a result of the time of sampling which in Cardinosa study was within the first 24 hours of admission but in our study the sample was done after the first 24 hours i.e. following extubation. In addition, McShane and Hone have showed coagulase-negative *Staphylococci* to be the most common source of infections among patients with ET (26). Amini *et al*. also conducted a descriptive study on distribution of isolated microorganisms from tracheal tube of ICU patients declaring that *S. aureus* (23.6%), *Klebsiella* spp. (23.3%), *Acintobacter* spp. (20.7%), *P. aeroginosa* (18.2%), *E. coli* (7.7%), and *Enterobacter* spp, were the most common isolates (27). Our study as well as the other ones in similar settings confirm that *Acinetobacter*, *P. aeruginosa*, and *S. aureus* are among the most 3 prevalent isolated organism from ET.

In contrast, Rello *et al*. in their study have demonstrated that *P. aeruginosa* is the most common causative organism for infection of ET and this could be the result of large number of patients with chronic obstructive pulmonary disease (COPD) and long time of intubation and even a previous history of antibiotic therapy (28). It should be noted that although there are minor variations in relative frequency of obtained organisms, different studies have reported varying sources of isolations as in our study we did report colonization of organisms into the ET tube while other studies may have considered ET-related infections in their study analysis.

In a study by Nazal –Matunog, most of the Gram negative bacteria were sensitive to ciprofloxacin compared with 3% resistant cases; there was amikacin resistance in 9.7% of the cases with the highest resistance to cefamandole (57%), cefotaxime (50%), and tobramycin (50%) (29). Khosravi *et al*. showed that *Enterobacter* spp. was the most prevalent organism colonized the endotracheal tubes (41.14%). It was followed by *Pseudomonas aeruginosa* (15.35%), *E. coli* (13.97.2%), coagulase negative staphylococci (14.76%), *S. aureus* (13.97%), and *Proteus* spp (0.79%) (30). Antimicrobial testing also revealed that the most resistant Gram negative isolate is *P. aeruginosa* with highest resistance to cefixime (70.8%), and coagulase negative staphylococci as the most resistant Gram positive isolates with highest resistance to oxacillin (84.2%). The concern which is similarly subject to all the studies is the increasing prevalence of resistant *P. aeruginosa* in critically ill patients which may lead to significant mortality as well as huge widespread to other wards of the hospital. In a study by Semoni *et al*. all of the included samples consisted of more than 1 pathogen while in our study each culture has only one pathogen. However, in the same line with our results, all of the organisms in their study were aerobics including *Streptococcus viridance*, *S. aureus* and *P. aeruginosa* (23).

One of the limitations of this study was the fact that although antibiotic resistance pattern is important to intensive care physician, this is also heavily influenced by the antibiotic usage pattern in the study patients prior to obtaining the sample and also overall antibiotic usage pattern in the study institution. In this regard, the utility of the reported results to other centers is limited.

In conclusion, this study presents the most common microorganisms colonized from endo-tracheal tube of hospitalized patients and their pattern of antibiotic resistance. As our study showed, *Acinetobacter* is the most common microorganism isolated from endotracheal tube. Ciprofloxacin was also the most prevalent antibiotic revealing resistant pattern. Moreover, most of the microorganisms were sensitive to imipenem and pipracillin-tazobactam.
